# Curbside particulate matter and susceptibility to SARS–CoV-2 infection

**DOI:** 10.1016/j.jacig.2023.100141

**Published:** 2023-11

**Authors:** Lisa Miyashita, Gary Foley, Sean Semple, Joseph M. Gibbons, Corinna Pade, Áine McKnight, Jonathan Grigg

**Affiliations:** a Blizard Institute, Queen Mary University of London, London, United Kingdom; b Institute for Social Marketing, University of Stirling, Stirling, United Kingdom

**Keywords:** Particulate matter, PM_10_, SARS-CoV-2, airway epithelial cells

## Abstract

**Background:**

Biologic plausibility for the association between exposure to particulate matter (PM) less than 10 μm in aerodynamic diameter (PM_10_) and coronavirus disease 2019 (COVID-19) morbidity in epidemiologic studies has not been determined. The upregulation of angiotensin-converting enzyme 2 (ACE2), the severe acute respiratory syndrome coronavirus type 2 (SARS-CoV-2) entry receptor on host cells, by PM_10_ is a putative mechanism.

**Objective:**

We sought to assess the effect of PM_10_ on SARS-CoV-2 infection of cells *in vitro*.

**Methods:**

PM_10_ from the curbside of London's Marylebone Road and from exhaust emissions was collected by cyclone. A549 cells, human primary nasal epithelial cells (HPNEpCs), SARS-CoV-2–susceptible Vero-E6 and Calu3 cells were cultured with PM_10_. ACE2 expression (as determined by median fluorescent intensity) was assessed by flow cytometry, and ACE2 mRNA transcript level was assessed by PCR. The role of oxidative stress was determined by N-acetyl cysteine. The cytopathic effect of SARS-CoV-2 (percentage of infection enhancement) and expression of SARS-CoV-2 genes' open reading frame (ORF) 1ab, S protein, and N protein (focus-forming units/mL) were assessed in Vero-E6 cells. Data were analyzed by either the Mann-Whitney *U* test or Kruskal-Wallis test with the Dunn multiple comparisons test.

**Results:**

Curbside PM_10_ at concentrations of 10 μg/mL or more increased ACE2 expression in A549 cells (*P* = .0021). Both diesel PM_10_ and curbside PM_10_ in a concentration of 10 μg/mL increased ACE2 expression in HPNEpCs (*P* = .0022 and *P* = .0072, respectively). ACE2 expression simulated by curbside PM_10_ was attenuated by N-acetyl cysteine in HPNEpCs (*P* = .0464). Curbside PM_10_ increased ACE2 expression in Calu3 cells (*P* = .0256). In Vero-E6 cells, curbside PM_10_ increased ACE2 expression (*P* = .0079), ACE2 transcript level (*P* = .0079), SARS-CoV-2 cytopathic effect (*P* = .0002), and expression of the SARS-CoV-2 genes' ORF1ab, S protein, and N protein (*P* = .0079).

**Conclusions:**

Curbside PM_10_ increases susceptibility to SARS-COV-2 infection *in vitro*.

Epidemiologic studies report an association between exposure to air pollution and susceptibility to coronavirus disease 2019 (COVID-19) due to severe acute respiratory syndrome coronavirus 2 (SARS-CoV-2) infection. For example, Zhu et al^1^ reported that a 10-μg/m^3^ increase in particulate matter (PM) less than 2.5 μm (PM_2.5_) over the previous 2 weeks is associated with a 2.2% increase (95% CI = 1.02-3.4) in cases of newly confirmed COVID-19, and Fattorini and Regoli^2^ reported a significant correlation between long-term air quality data from Italian provinces and COVID-19 cases. More recently, in a retrospective analysis of SARS-CoV-2–positive patients, Bozack et al^3^ reported that higher long-term exposure to inhalable PM is associated with increased COVID-19 mortality, and Chen et al^4^ reported an association between exposure to ambient PM_2.5_ and both COVID-19 severity and mortality in individuals who received diagnoses of COVID-19 from Kaiser Permanente Southern California.

The host receptor for SARS-CoV-2 infection is angiotensin-converting enzyme 2 (ACE2). The ACE2 receptor is expressed by upper and lower airway epithelial cells and organs such as the kidney and gut.^5^, ^6^, ^7^ Cells susceptible to SARS-CoV-2 infection *in vitro* include the monkey kidney–derived cell line Vero-E6 and human lung cancer–derived Calu3 cells.^8^ The Vero-E6 cell line is the cell line used most widely in infection studies because it both expresses ACE2 and exhibits a prominent and reproducible cytopathic effect (CPE).^9^^,^^10^

There is indirect evidence from previous mechanistic studies that fossil fuel–derived PM increases susceptibility of airway cells to SARS-CoV-2 infection. First, Sagawa et al^11^ reported that intratracheal instillation of urban ambient PM increases murine ACE2 expression by alveolar type 2 (AT2) cells. Second, Zhu and et al^12^ reported that in a murine model, urban PM increases lung ACE2 and subsequent SARS-CoV-2 viral load. In human cells, Kim et al^13^ reported that diesel PM_2.5_ increases ACE2 expression and ACE2 transcript level in pluripotent stem cell–derived airway epithelial cells, and Zhu et al^12^ reported that urban PM increases ACE2 protein expression in pulmonary alveolar epithelial cells *in vitro*. To date, the effect of PM_10_ on ACE2 expression by human nasal epithelial cells (the initial site of SARS-CoV-2 infection^5^^,^^14^) and the functional relevance of PM-induced changes in ACE2 are unclear.

In this study, we sought to assess the effect of curbside PM_10_ on ACE2 expression in human nasal epithelial airway cells, and in Vero-E6 cells we sought to determine whether curbside PM_10_ increases susceptibility to SARS-CoV-2 infection. The effect of cigarette smoke extract (CSE) was also assessed because it is a source of oxidative stress,^15^ it increases ACE2 expression in primary airway epithelial cells *in vitro*,^16^ and ACE2 expression is increased in the lower airway of smokers.^17^

## Methods

### Collection of PM_10_

Curbside PM_10_ was collected using a high-volume cyclone placed at a single site on the curbside of Marylebone Road, one of the most polluted roads in London.^18^ Sampling was done for 6 to 8 hours per day on 11 occasions. Diesel exhaust PM_10_ was collected by placing the cyclone 0.1 m from the end of the tailpipe of an idling diesel car (BMW, model d330) for 15 minutes. The PM_10_ was stored at room temperature in a sterile glass container. Aliquots of the diesel PM_10_ and curbside PM_10_ were diluted in Dulbecco PBS to a final concentration of 1 mg/mL and stored as a master stock at –20°C.

### CSE

CSE was prepared as previously reported.^19^ Briefly, cigarette smoke in filter material was prepared by using 3 cigarettes (0.8 mg of nicotine per cigarette; Marlboro, Philip Morris USA, Pittsburgh, Pa) "smoked" by a water aspirator, with the cigarette smoke aspirated through a cotton wool filter. CSE (100%) was obtained by vortexing the cotton wool filter in 2 mL of dimethyl sulfoxide diluted in medium.

### Cells

The human AT2 epithelial cell line A549 was purchased from Sigma-Aldrich (Poole, UK) and maintained in Dulbecco modified eagle medium (DMEM) supplemented with FBS and penicillin-streptomycin (Lonza, Basel, Switzerland). The passage number was less than 20, with the same number of passages used in each set of experiments. Human primary nasal epithelial cells (HPNEpCs) were either obtained from a nonsmoking, nonvaping, healthy adult donor by using a dental brush (donor HPNEpCs) or purchased from PromoCell (Heidelberg, Germany). The nasal cells were maintained in airway epithelial cell growth medium (AECGM) by using a PromoCell supplement kit (Heidelberg, Germany) with Primocin (InvivoGen, San Diego, Calif) and stored cryogenically in freezing medium (AECGM: 10% FBS, 10% dimethyl sulfoxide) at passage 1. A vial of HPNEpCs was thawed and aliquoted into multiple T25 cell culture flasks (VWR International, Lutterworth, UK). The cells were maintained in AECGM until confluent. The passage number was less than 2. Vero-E6 cells were obtained from the American Type Culture Collection (ATCC, Middlesex, UK) and maintained in DMEM supplemented with FBS and penicillin-streptomycin (Lonza, Basel, Switzerland). The passage number was less than 20. Calu3 cells were purchased from ATCC maintained in Eagle minimum essential medium (ATCC) supplemented with FBS (ATCC) and penicillin-streptomycin (Lonza). The passage number was less than 5. Cell membrane integrity was assessed by lactate dehydrogenase (LDH) release, according to the manufacturer’s instructions (Sigma-Aldrich). Treatment of cells with distilled water was used as a positive control and indicated 100% LDH release. For exposure to PM_10_, master stock PM_10_ was thawed, thoroughly vortexed, and suspended at a final concentration of 1 to 20 μg/mL in Dulbecco PBS (2% FBS) containing a 2 × 10^5^-cell monolayer per well for 2 hours at 37 °C. Control samples of medium (ie, medium without without PM_10_) were incubated with the same volume of Dulbecco PBS (2% FBS). In some experiments, the antioxidant N-acetyl cysteine was added with PM_10_ at a final concentration of 5 mmol·L.

### ACE2 expression by flow cytometry

Cells were washed twice and stained with either anti-ACE2 (Abcam, Cambridge, UK; catalog no. ab272690) or isotype control primary antibodies (Abcam; catalog no. Ab171870) for 1 hour at room temperature. The epithelial cell marker E-cadherin (Abcam; catalog no. Ab1416) was included in all reactions. The cells were then washed and stained with secondary antibodies conjugated to Alex Fluor 488 for ACE2/isotype expression (Abcam; catalog no. Ab150077), or allophycocyanin for E-cadherin expression (Abcam; catalog no. Ab130786) for 30 minutes in the dark at room temperature. The cells were finally washed, and ACE2 expression measured by using az BD FACS Canto II flow cytometer (BD Biosciences, San Diego, Calif). E-cadherin–positive cells were selected to exclude cell debris, and the mean fluorescence intensity of fluorescein isothiocyanate was calculated with adjustment for the isotypic control.

### ACE2 expression by qRT-PCR

The cells were washed and total RNA was extracted by using the RNeasy mini kit (Qiagen, Manchester, UK) according to the manufacturer’s instructions. cDNA was synthesized from total RNA by using Superscript II (Thermo Fischer cell culture flasks [VWR International]). Quantitative RT-PCR (qRT-PCR) was performed by using custom TaqMan array 96-well plates and TaqMan Fast Universal Master mix II with UNG (Life Technologies) with ACE2 and the housekeeping gene glyceraldehyde 3-phosphate dehydrogenase (*GAPDH*). Amplification was performed with the fast PCR 7500 (Life Technologies). The cycling conditions started with an initial activation step for 15 minutes at 95°C, followed by 40 cycles of denaturation for 15 seconds at 94°C, annealing for 30 seconds at 55°C, and extension for 30 seconds at 70°C, with fluorescence data collection performed during the extension step. The threshold cycle values were normalized to *GAPDH*, which is a frequently used reference gene, and relative quantification was analyzed by using the 2^-ΔΔCt^ method.

### Preparation and titration of authentic SARS-CoV-2

Wuhan Hu-1 strain SARS-CoV-2 (2019-nCoV/BavPat1/2020) authentic virus cell culture supernatant (isolate collection date January 1, 2020) was obtained from the European Virus Archive Global (EVAg, Charité Universitätsmedizin Berlin, Germany). Virus stocks were prepared as previously described.^20^^,^^21^ Vero-E6 cells were seeded in 75-cm^2^ cell culture flasks 24 hours before inoculation with virus cell culture supernatant containing 2.2 × 10^6^ plaque forming units (PFU) in a volume of 10 mL of DMEM 10% FBS. The flasks were observed daily, and virus-containing cell culture medium was harvested when more than 80% of the cells showed CPE. The supernatant was centrifuged at 500 *g* for 5 minutes to clear cell debris and aliquots stored at –80°C. To determine the titer of SARS-CoV-2 virus stocks, Vero-E6 cells were seeded in 48-well plates at a rate of 3×10^4^ cells per well. After 24 hours, adherent cell monolayers were challenged with serial 1:10 duplicate dilutions of virus, and titer was assessed after 20 hours by *in situ* intracellular staining to identify foci of infection. The cells were washed in PBS, fixed in ice-cold methanol-acetone (50:50), and virus antigen was stained for 1 hour at 37°C by using sera from convalescent individuals diluted 1:2000 in PBS (1% FCS). The cells were washed a further 3 times in PBS and incubated with goat anti-human IgG β-galactosidase–conjugated antibody (catalog no. 2040-06, Southern Biotech, Birmingham, Ala) diluted 1:400 in PBS (1% FCS) for 1 hour at 37°C. After 3 further PBS washes, 300 μL of 0.5-mg/mL 5-bromo-4-chloro-3-indolyl ß-D-galactopyranoside chromogenic substrate (X-gal) in PBS containing 3 mM potassium ferricyanide, 3 mM potassium ferrocyanide, and 1 mM magnesium chloride was added to each well. The infected cells incubated at 37°C stained blue within 1 to 4 hours after addition of substrate, and the clusters of blue cells were counted as foci of infection to determine the virus titer defined as focus-forming units (FFU)/mL. All authentic SARS-CoV-2 propagation and infectivity assays were performed in a containment level 3 facility.

### SARS-CoV-2 infection

Vero-E6 cells were suspended in DMEM-supplemented FBS and penicillin-streptomycin overnight in adherent 46-well cell culture plates at 37°C. The cells were then washed and cultured with PM_10_ or control medium for 2 hours before being thoroughly washed and then challenged with 3 × 10^4^ FFU/mL of SARS-CoV-2 (the median tissue culture infectious dose) in duplicate and incubated at 37°C for 48 hours to 72 hours. The surviving cells were fixed in 3.7% (vol/vol) formaldehyde and stained with 0.1% (wt/vol) crystal violet solution. The crystal violet stain was resolubilized in 1% (wt/vol) SDS. Absorbance readings were taken at 57 nm by using a CLARIOStar Plate Reader (BMG Labtech, Aylesbury, UK). The absorbance readings were normalized to SARS-CoV-2 virus–only control wells, and enhancement/inhibition in infection was presented as the percentage change in CPE (percent enhancement of infection).

### Viral gene expression by qRT-PCR

qRT-PCR was performed by using the TaqMan 2019-nCoV Assay Kit v1 and TaqPath 1-Step RT-qPCR Master Mix (A47532 and A15299, Thermo Fisher). The kit includes 3 primer sets that target SARS-CoV2 genes' open reading frames (ORF) 1ab, spike (S) protein, and nucleocapsid (N) protein. A standard curve was generated with an input of 1.5 × 10^6^ FFU/mL, and serial 10-fold dilutions of SARS-CoV-2 viral stock were made. Viral RNA was then extracted from the stock to generate a standard curve. The cells were challenged with SARS-CoV-2 (Wuhan Hu-1) for 48 hours before the cell supernatants were removed and viral RNA was extracted from the cell lysates. Amplification was performed with the fast PCR 7500 (Life Technologies). The cycling conditions started with an initial hold stage for 2 minutes at 25°C, reverse transcription for 15 minutes at 50°C, and an activation stage for 2 minutes at 95°C, followed by 40 cycles of denaturation for 3 seconds at 94°C and annealing/extension for 30 seconds at 60°C, with fluorescence data collection being performed during the extension step. The threshold cycle values were normalized to the standard curve for quantification, and the results were expressed as FFU/mL.

### Ethics

Sampling of nasal epithelial cells was approved by the Queen Mary University of London ethics committee (reference no. 15/NE/0237).

### Statistical analysis

Data are summarized as medians and analyzed by either the Mann-Whitney *U* test or Kruskal-Wallis test with the Dunn multiple comparisons test. The data are from at least 4 separate experiments. The analysis were performed by using Prism 9.0, with *P* values less than .05 considered statistically significant. Statistical analysis was performed by using GraphPad Prism 9.0 (GraphPad Software, La Jolla, Calif).

## Results

**Fig 1:**
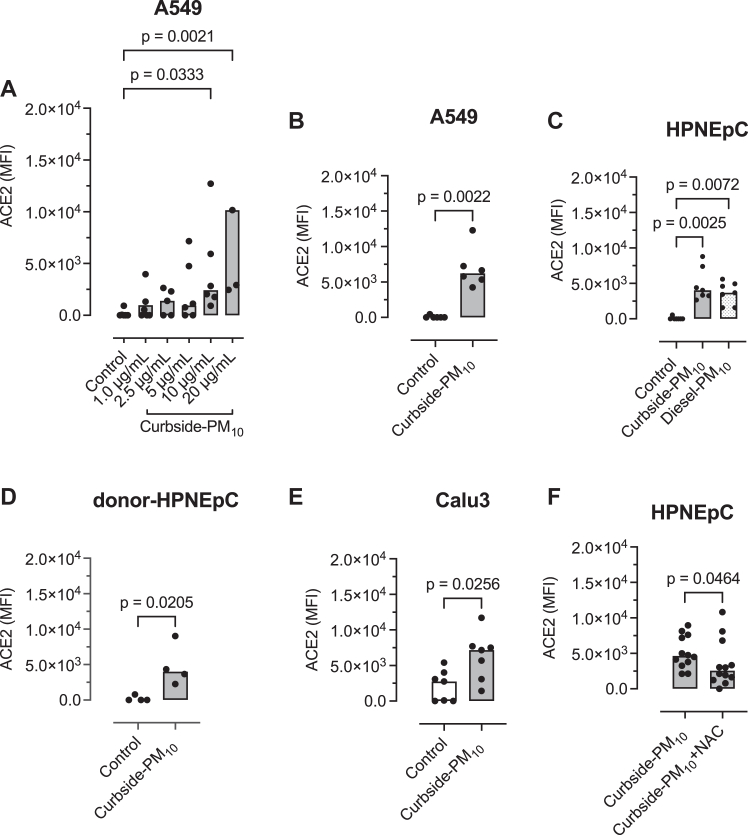
Effect of PM_10_ on ACE2 expression. PM_10_ was collected from the curbside of Marylebone Road (curbside PM_10_) and the exhaust of a diesel car (diesel PM_10_) by using the same cyclone. Cells were cultured with PM_10_ for 2 hours, and ACE2 expression was determined by flow cytometry. The results are expressed as mean fluorescence intensity (MFI) adjusted for isotypic antibody control. **A,** Dose-response relationship between curbside PM_10_ and ACE2 expression by A549 cells. Two data points are omitted for visual convenience. **B**, Replication of A549 dose-response data using 10 μg/mL of curbside PM_10_. **C**, Effect of curbside PM_10_ and diesel PM_10_ (10 μg/mL) on ACE2 expression (MFI) in purchased (Promocell) HPNEpCs. **D,** Effect of curbside PM_10_ (10 μg/mL) on ACE2 expression in primary nasal epithelial cells obtained from a local adult donor HPNEpCs. **E,** Effect of curbside PM_10_ (10 μg/mL) on ACE2 expression (MFI) in Calu3 cells. **F,** Effect of the antioxidant N-acetyl cysteine on expression of curbside PM_10_-stimulated ACE2 expression (MFI) in purchased HPNEpCs. Columns represent medians from at least 4 separate experiments. Data were compared by either the Mann-Whitney *U* test or Kruskal-Wallis test and Dunn multiple comparisons test.

In the dose-response experiment, curbside PM_10_ in a concentration of 10 μg/mL or higher increased ACE2 expression in A549 cells (*P* = .0021 [Fig 1, *A*]). In the time-course experiment, curbside PM_10_ in a concentration of 10 μg/mL increased ACE2 expression at 2 hours or more in A549 cells (*P* = .0003 [see Fig E1 in the Online Repository at www.jaci-global.org]). Culturing A549 cells with 10 μg/mL of curbside PM_10_ for 2 hours did not impair cell membrane integrity assessed by LDH release (see Fig E2 in the Online Repository at www.jaci-global.org); this concentration and duration were used in subsequent experiments.

In A549 cells, a single concentration of 10 μg/mL of curbside PM_10_ increased ACE2 expression (*P* = .0022 [Fig 1, *B*]). In purchased HPNEpCs (Promocell), diesel exhaust PM_10_ (10 μg/mL) and curbside PM_10_ (10 μg/mL) significantly increased ACE2 expression (*P* = .0025 and *P* = .0072, respectively [Fig 1, *C*]). In HPNEpCs from a local adult donor, curbside PM_10_ (10 μg/mL) increased ACE2 expression (*P* = .0205 [Fig 1, *D*]). In SARS-CoV-2–susceptible Calu3 cells, curbside PM_10_ (10 μg/mL) stimulated ACE2 expression (*P* = .0256 [Fig 1, *E*]), and in purchased HPNEpCs, the antioxidant N-acetyl cysteine attenuated ACE2 expression stimulated by curbside PM_10_ (*P* = .0464 [Fig 1, *F*]).

**Fig 2:**
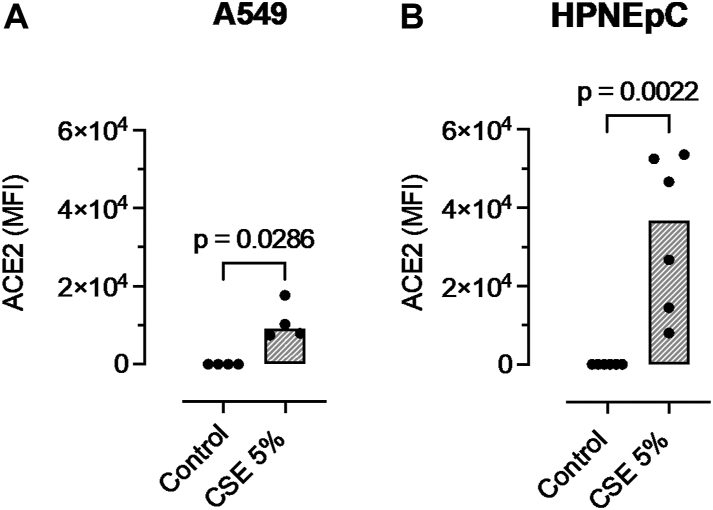
Effect of 5% CSE on ACE2 expression in A549 cells (**A**) and purchased (from Promocell) HPNEpCs (**B**). Results are expressed as mean fluorescence intensity (MFI) adjusted for isotypic antibody control. Columns represent medians from at least 4 separate experiments, and data are compared by the Mann-Whitney *U* test.

In both A549 cells and purchased-HPNEpCs (Promocell), CSE 5% increased ACE2 expression (*P* = .0286 and *P* = .0022, respectively, vs the control [Fig 2, *A* and *B*]). In purchased HPNEpCs, ACE2 expression stimulated by CSE 5% was increased compared with curbside PM_10_ (median mean fluorescence intensity = 36663 [interquartile range = 12886-52782] vs median mean fluorescence intensity = 4046 [interquartile range = 3258-7406]; *P* = .002).

**Fig 3:**
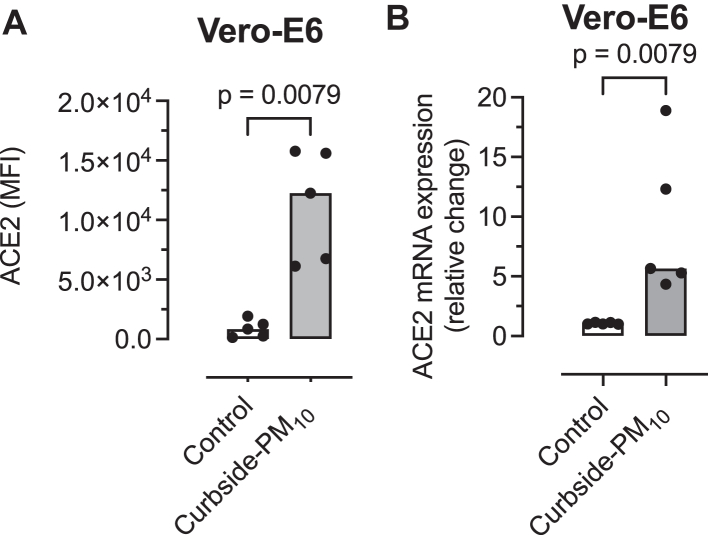
Effect of curbside PM_10_ (10 μg/mL) for 2 hours on Vero-E6 cell. **A,** ACE2 expression by flow cytometry expressed as mean fluorescence intensity (MFI) adjusted for isotypic antibody control. **B,** ACE2 transcript level. Threshold cycle values were normalized to glyceraldehyde 3-phosphate dehydrogenase, and relative quantification was analyzed by using the 2^-ΔΔCt^ method. Columns represent medians from 5 separate experiments, and data are compared by the Mann-Whitney *U* test.

**Fig 4:**
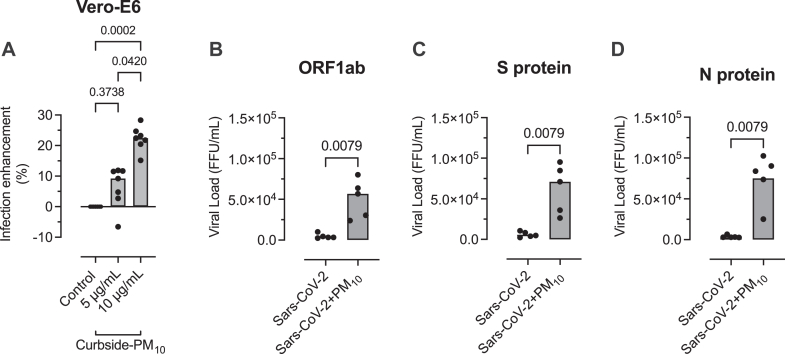
Effect of curbside PM_10_ on SARS-CoV-2 infection of Vero-E6 cells. **A,** SARS-CoV-2 CPE expressed as infection enhancement (percentage) compared with the control medium. Data from 7 separate experiments are compared by the Kruskal-Wallis test and Dunn multiple comparisons test. Effect of curbside PM_10_ on SARS-CoV-2 viral gene expression in Vero-E6 cells: ORF 1ab (**B**), S protein (**C**), and N protein (**D**). Viral protein data from 5 separate experiments are expressed as FFU/mL calculated from a standard curve with an input of 1.5 × 10^6^ FFU/mL and serial 10-fold dilutions of SARS-CoV-2 viral stock. Ct values are normalized to the standard curve for quantification. Columns represent medians from 5 separate experiments, and data are compared by using the Mann-Whitney *U* test.

In Vero-E6 cells, curbside PM_10_ (10 μg/mL) increased ACE2 expression (*P* = .0079 [Fig 3, *A*]) and ACE2 transcript level (*P* = .0079 [Fig 3, *B*]). In Vero-E6 cells exposed to curbside PM_10_ and then infected with SARS-COV-2, there was a dose-dependent increase in CPE (percent enhancement of infection = 5 μg/mL vs control [*P* = .3738], 10 μg/mL vs control [*P* = .0002], and 5 μg/mL vs 10 μg/mL [*P* = .042] [Fig 4, *A*]). Curbside PM_10_ (10 μg/mL) in Vero-E6 cells increased SARS-CoV-2 gene (FFU/mL) ORF1ab (*P* = .0079 [Fig 4, *B*]), S protein (*P* = .0079 [Fig 4, *C*]), and N protein (*P* = .0079 [Fig 4, *D*]).

## Discussion

In this study, expression of ACE2 (the receptor coopted by SARS-CoV-2 to infect cells^22^) by human nasal epithelial cells was stimulated by PM_10_ collected from the curbside of a London main road dominated by diesel traffic.^23^ PM_10_ stimulation of ACE2 expression was also found in A549 cells, a cell line that is widely used to model AT2 cell responses^24^ and normally expresses very low to absent ACE2.^10^ Consistent with these A549 data, Sagawa et al^11^ reported increased ACE2 protein expression in murine AT2 cells after intratracheal instillation of 500 μg of urban PM,^11^ Kim et al^13^ reported increased ACE2 expression by human pluripotent stem cell–derived airway cells cultured *in vitro* with 50 μg/mL diesel PM_2.5_ for 48 hours, and Zhu et al^12^ reported increased ACE2 protein expression in human pulmonary alveolar epithelial cells exposed to 50 μg/mL of urban PM for 24 hours. In the present study, the functional relevance of PM_10_-stimulated ACE2 was established in Vero-E6 cells. In this SARS-CoV-2 trophic cell line, curbside PM_10_ increases ACE2 expression and ACE2 transcript levels and enhances SARS-CoV-2 infection and SARS-COV-2 viral gene expression.

To date, the pathway by which PM_10_ simulates ACE2 expression is unclear. However, Santurtún et al^25^ speculated that the initial stimulus is PM-induced oxidative stress. Compatible with this speculation, we found that the antioxidant N-acetyl cysteine attenuated PM_10_-stimualted ACE2 expression. Furthermore, we found that CSE, a known source of oxidative stress,^15^^,^^16^ increased ACE2 expression in both A549 cells and HPNEpCs. Indeed, in HPNEpCs, CSE 5% was the more potent stimulus of ACE2 expression. We therefore speculate that smokers living in areas with high concentrations of PM_10_ are more vulnerable to SARS-CoV-2 infection.

There are limitations to this study. First, because the PM concentration in nasal epithelial lining fluid is unknown, whether exposure of cells to 10 μg/mL PM_10_ *in vitro* reflects exposure *in vivo* is unclear even though concentrations of PM_10_ on the curbside of Marylebone Road may exceed 180 μg/m^3^.^26^ In addition, Zielinski et al^27^ have calculated that 50 μg/mL of PM in *in vitro* experiments likely reflects PM concentrations in the human airway epithelial lining fluid when individuals are exposed to the maximum UK hourly average value. Second, the SARS-CoV-2 infection experiments were performed in nonhuman Vero-E6 cells because in contrast to primary airway epithelial cells, this cell line is permissive for SARS-CoV-2 infection, resulting in a highly reproducible CPE. Third, we did not assess the expression of other proteins that support SARS-CoV-2 infection. For example, SARS-CoV-2 uses transmembrane protease serine 2 (TMPRSS2) to prime its spike protein, although Vero-E6 cells express TMPRSS2 at very low levels.^8^ Fourth, the demographic details of the purchased human primary nasal epithelial cell donor are unknown. However, we replicated the ACE2 response of purchased cells by using primary nasal cells from a local adult donor. Fifth, Crilley et al^28^ found elements in PM from Marylebone Road that are indicative of abrasion processes from tyres and brakes. Although we cannot exclude upregulation of ACE2 by PM from these noncombustion sources, diesel PM is likely to be a major source of curbside PM_10_ in the present study because when using particle-induced x-ray emission analysis, Crilley et al^28^ found that diesel emissions are the main source of curbside PM on Marylebone Road, and we confirmed that diesel PM_10_ per se upregulates ACE2 expression. Finally, whether PM_10_ increases susceptibility of nasal epithelial cells to other infections is unclear. However, Takizawa et al^29^ reported that diesel exhaust PM upregulates intercellular adhesion molecule-1 (ICAM-1), the entry receptor for rhinoviruses,^30^ in human bronchial epithelial cells *in vitro*.

In summary, PM_10_ sampled from the curbside of a main road in London via induction of oxidative stress upregulates ACE2 expression by airway epithelial cells *in vitro*. This in turn increases vulnerability to SARS-CoV-2 infection. Thus, the association between exposure to fossil fuel–derived PM_10_ and susceptibility to COVID-19 that has been reported in epidemiologic studies is biologically plausible.

### Data sharing

Data collected for the study and a data dictionary defining each field in the set will be made available without restriction. These data will be available with publication. Data as original Graph Pad prism files will be made available and may be obtained by e-mailing the corresponding author (j.grigg@qmul.ac.uk). Some data described are presented as preprints available at the following sites: https://doi.org/10.2139/ssrn.4285489 and https://www.biorxiv.org/content/10.1101/2020.05.15.097501v1.

## Disclosure statement

Supported by the Barts Charity (grant MGU0547), the 10.13039/501100000265Medical Research Council (grant MR/R009848/01), the Saint Bartholomew's Hospital Trust (grant 17/LO/1752), and the 10.13039/501100000833Rosetrees Trust (grant CF1100003).

Disclosure of potential conflict of interest: J. Grigg provided medical advice to a UK inquest at which air pollution was identified a cause of death of an asthmatic child. J. Grigg is supported by a NIHR Senior Investigator Award. The rest of the authors declare that they have no relevant conflicts of interest.

## Supplementary data

Supplementary Figures E1 and E2
