# Supplementary material for: Curbside particulate matter and susceptibility to SARS–CoV-2 infection
Source: J Allergy Clin Immunol Glob. 2023 Nov;2(4):100141. doi: 10.1016/j.jacig.2023.100141 (PMC10509961; doi:10.1016/j.jacig.2023.100141)
Supplement: Supplementary Figures E1 and E2 [file mmc1.docx]

**Curbside particulate matter less than 10 microns in aerodynamic diameter and susceptibility to SARS-COV-2 infection *in vitro***

**Figure E1**

Time-course for the effect of particulate matter less then 10 microns in aerodynamic diameter (PM_10_) on angiotensin-converting enzyme 2 (ACE2) expression in A549 cells. Cells were cultured with curbsidePM_10_ 10 μg/mL for 0.5 to 4 h, and ACE2 expression determined by flow cytometry. Data are expressed as median fluorescent intensity (MFI) adjusted for isotypic antibody control. Column represent median from 5 separate experiments and compared by Kruskal-Wallis test and Dunn’s multiple comparisons test.

**Figure E2**

Effect of curbside-PM_10_ on lactate dehydrogenase (LDH) release from A549 cells. Cells were incubated with curbside-PM_10_ 10 µg/mL for 2 h. Data are indexed to LDH release after lysis with distilled water. Column represents median of 2 separate experiments.
